# Feature Selection and Machine Learning Strategies for CT Radiomics-Based Survival Prediction in Non-Small Cell Lung Cancer: A Comparative Study †

**DOI:** 10.3390/diagnostics16121761

**Published:** 2026-06-07

**Authors:** Mohan Huang, Ashley Hui, Ching Wai Leung, Chun Lam Li, Tsz Lung Leung, Fuk-Hay Tang, Shing Yau Tam

**Affiliations:** School of Medical and Health Sciences, Tung Wah College, Homantin, Hong Kong SAR, China; mhhuang@twc.edu.hk (M.H.); fhtang@twc.edu.hk (F.-H.T.)

**Keywords:** non-small cell lung cancer, radiomics, computed tomography, machine learning, feature selection, survival prediction, prognosis

## Abstract

**Background/Objectives**: Computed tomography (CT)-based radiomics shows promise for non-small cell lung cancer (NSCLC) prognosis prediction, but model performance varies widely by feature selection and machine learning strategies. Optimal combinations remain unclear. This study aims to systematically compare feature selection methods and machine learning algorithms for 12-month overall survival prediction using CT radiomics in NSCLC patients. **Methods**: We analyzed 385 patients from The Cancer Imaging Archive (TCIA) NSCLC-Radiomics dataset. Radiomic features from primary tumor volumes were combined with clinical variables. Three feature selection methods—sequential forward selection (SFS), maximum relevance minimum redundancy (mRMR), and least absolute shrinkage and selection operator (LASSO)—were compared across five classifiers: k-nearest neighbors (KNN), support vector machine (SVM), random forest (RF), logistic regression (LR), and gradient boosting classifier (GBC). Performance was assessed using area under the receiver operating characteristic curve (AUC) and accuracy on independent test sets. Cox regression and Kaplan–Meier analyses evaluated survival risk stratification. **Results**: Logistic regression showed the most stable classification performance across feature selection strategies (test AUC 0.60–0.65, accuracy 0.72–0.73). The mRMR-LR model achieved highest AUC (0.65); LASSO-LR showed highest accuracy (0.73). For survival analysis, LASSO-based Cox modeling demonstrated superior risk stratification with significant separation between high- and low-risk groups in both training and testing sets (*p* = 0.0095). **Conclusions**: Simpler models like logistic regression provide robust performance in CT radiomics, while LASSO excels for survival risk stratification. As we employed single-dataset validation, clinical applicability remains limited because validation was performed within a single public dataset. Nevertheless, the findings provide methodological insights into the selection of feature selection and machine learning strategies for CT radiomics-based prognostic modeling in NSCLC.

## 1. Introduction

Lung cancer remains one of the most frequently diagnosed malignancies and the leading cause of cancer-related mortality worldwide. Among all lung cancer subtypes, non-small cell lung cancer (NSCLC) accounts for approximately 85% of cases and exhibits substantial heterogeneity in clinical outcomes [1]. Despite continuous advances in surgical techniques, radiotherapy, systemic therapies, and immunotherapy, survival outcomes among NSCLC patients remain highly variable, even within the same clinical stage [2]. This highlights the limitation of conventional clinicopathological staging systems in accurately capturing tumor biological heterogeneity and predicting patient prognosis.

Medical imaging, particularly computed tomography (CT), plays a central role in the diagnosis, staging, treatment planning, and follow-up of NSCLC. In recent years, radiomics has emerged as a powerful quantitative imaging approach that enables the extraction of high-dimensional features from standard-of-care medical images. These features can characterize tumor intensity, shape, texture, and intratumoral heterogeneity in a non-invasive manner [3,4,5]. Previous studies have demonstrated that CT-based radiomic features are associated with tumor phenotype and clinical outcomes, suggesting their potential as imaging biomarkers for prognostic assessment [6,7].

However, the development of radiomics-based predictive models faces several methodological challenges. Radiomic datasets are typically characterized by high dimensionality and relatively small sample sizes, making them particularly susceptible to overfitting [8,9]. As a result, feature selection plays a critical role in identifying informative and robust features while reducing redundancy. Various feature selection methods, such as sequential forward selection (SFS), maximum relevance minimum redundancy (mRMR), and least absolute shrinkage and selection operator (LASSO), have been widely applied in radiomics studies [9,10,11,12]. Meanwhile, different machine learning algorithms, including both linear and non-linear models, may exhibit distinct performance characteristics when applied to radiomics data [13].

Although numerous studies have proposed radiomics-based models for survival prediction in NSCLC, several critical clinical gaps remain unaddressed. Most existing studies focus on optimizing a single modeling pipeline, making it difficult to determine whether the performance is attributable to the feature selection strategy, the machine learning methods, or their specific interaction. Consequently, there remains a lack of systematic evaluation comparing how different combinations of feature selection strategies and machine learning algorithms influence predictive performance and generalizability on the same dataset. In particular, it is unclear whether more complex machine learning models consistently outperform simpler approaches in high-dimensional radiomics settings [14]. Consequently, researchers and clinicians lack practical guidance on which modeling strategies are most robust for CT radiomics-based survival prediction in NSCLC.

To address this gap, this study makes the following technical contributions. Our study aims to systematically evaluate the impact of multiple feature selection methods and machine learning algorithms on CT radiomics-based prediction of 12-month overall survival in NSCLC patients. Specifically, three feature selection strategies (SFS, mRMR, and LASSO) were combined with five machine learning algorithms (k-nearest neighbors (KNN), support vector machine (SVM), random forest (RF), logistic regression (LR) and gradient boosting classifier (GBC)) to construct and compare predictive models. This study provided a quantitative assessment of how different feature selection strategies interact with different machine learning algorithms, identifying which combinations yield the most robust predictive performance within the studied dataset. In addition, survival analysis based on Cox proportional hazards models was performed to assess the prognostic stratification ability of the selected feature sets. By providing a comprehensive comparison across different modeling strategies, this study seeks to identify robust methodological strategies for radiomics-based survival prediction and provide practical guidance for future model development and validation studies in NSCLC.

## 2. Materials and Methods

### 2.1. Dataset

This retrospective study utilized data from The Cancer Imaging Archive (TCIA), a publicly available repository that provides de-identified medical imaging datasets for cancer research. Specifically, the NSCLC-Radiomics dataset available within TCIA was used, which originally included 422 patients diagnosed with non-small cell lung cancer (https://www.cancerimagingarchive.net/collection/nsclc-radiomics/ (accessed on 1 December 2024)) [15].

Clinical information included age, sex, TNM staging, overall stage, survival time, and survival status. Imaging data consisted of CT scans and associated radiotherapy (RT) structure sets, including gross tumor volume (GTV) segmentations.

Patients were excluded if they had missing RT structure sets, absence of GTV contours, incorrect segmentation (e.g., a single label including multiple tumors), inconsistencies between clinical and imaging data, or corrupted files.

After exclusion, a total of 385 patients were included in the final analysis. Patients censored before the 12-month endpoint were excluded because survival status at the predefined classification time point could not be determined (*n* = 37). The remaining patients were randomly allocated into training and testing sets at a ratio of 7:3 using a fixed random seed (random_state = 42) to ensure reproducibility. The primary tumor (GTV1) was selected for radiomic feature extraction in all patients to ensure consistency.

### 2.2. Radiomic Feature Extraction

The radiomic feature extraction included the following parts: (1) tumor segmentation; (2) imaging preprocessing; (3) radiomic features extraction.

Tumor regions of interest (ROIs) were manually segmented by two independent radiologists using 3D Slicer version. Any disagreements were resolved by consensus or by a third senior radiologist. Tumor segmentation was implemented in 3D slicer.

To reduce variability caused by different imaging acquisition protocols, all CT images were resampled to isotropic voxel spacing of 1 × 1 × 1 mm^3^ [16]. Gray-level discretization was performed using a fixed bin width of 25 Hounsfield units (HUs) to standardize intensity resolution.

Radiomic features were extracted from the primary tumor regions using the Pyradiomics extension implemented in 3D Slicer (Version 5.8.1) [17]. A total of 107 quantitative features were derived from each tumor volume, encompassing first-order statistics, texture features, and shape descriptors. Specifically, the extracted features included first-order intensity features, gray-level co-occurrence matrix (GLCM), gray-level dependence matrix (GLDM), gray-level run-length matrix (GLRLM), gray-level size zone matrix (GLSZM), neighborhood gray-tone difference matrix (NGTDM), and shape-based features. These features were designed to capture tumor intensity distribution, spatial heterogeneity, and morphological characteristics in a non-invasive manner. Feature extraction was performed following standardized radiomics workflows to ensure reproducibility and consistency across all samples.

### 2.3. Preprocessing

Clinical variables were encoded as follows: sex (male = 1, female = 0) and overall stage (I–IV encoded as 1–4). Missing values in clinical variables were handled using simple imputation, with the mean used for continuous variables (age) and the mode for categorical variables (overall stage). All continuous variables, including radiomic features and age, were standardized using z-score normalization based on the training set. The same transformation parameters (mean and standard deviation) were applied to the testing set to avoid data leakage.

### 2.4. Study Endpoint

This study selected 12-month overall survival as the primary outcome, treated as a binary variable. Patients censored within 12 months were excluded due to unknown survival status at the endpoint.

### 2.5. Feature Selection

To address the high dimensionality of radiomic features and reduce redundancy, three feature selection methods were applied: sequential forward selection (SFS), maximum relevance minimum redundancy (mRMR) [18], and least absolute shrinkage and selection operator (LASSO). These methods were selected due to their complementary characteristics in filtering, wrapper-based, and embedded feature selection approaches. Importantly, all feature selection procedures were conducted exclusively on the training set to prevent information leakage and ensure unbiased evaluation. The selected feature subsets were then applied to the testing set for independent performance evaluation.

### 2.6. Machine Learning Models

Five supervised machine learning algorithms were employed to construct predictive models for 12-month overall survival, including KNN, SVM, RF, LR, and GBC [19,20]. These models represent a combination of linear and non-linear approaches with varying levels of complexity. Each feature selection method was combined with all five algorithms, resulting in a total of 15 candidate models. Model training was performed using the training cohort, and performance was evaluated on an independent testing cohort. Default or commonly used parameter settings were adopted to ensure fair comparison across different algorithms.

### 2.7. Evaluation

Model performance was assessed using the area under the receiver operating characteristic curve (AUC) as the primary evaluation metric, complemented by accuracy. The AUC reflects the discriminative ability of the model across different classification thresholds, while accuracy provides an intuitive measure of classification performance. All evaluations were conducted on the independent testing set to ensure unbiased estimation of model performance. The model achieving the highest AUC was considered the optimal predictive model.

### 2.8. Survival Analysis

Survival analysis was conducted using the Cox proportional hazards model based on features selected by each feature selection method. Risk scores were calculated for each patient using the Cox model, and patients were stratified into high- and low-risk groups based on the median risk score in the training set. Kaplan–Meier survival curves were generated, and statistical differences between groups were assessed using the log-rank test. Survival analysis was performed on both training and testing sets to evaluate model robustness. All statistical analyses were conducted using Python (version 3.11.4).

## 3. Results

### 3.1. Patient Characteristics

A total of 385 patients were included in this study, with 269 patients assigned to the training set and 116 patients to the testing set. Baseline clinical characteristics between the two cohorts were compared to assess data distribution consistency. No statistically significant differences were observed between the training and testing sets in terms of age (*p* = 0.343), clinical T stage (*p* = 0.791), clinical N stage (*p* = 0.177), clinical M stage (*p* = 0.320), overall stage (*p* = 0.875), or sex (*p* = 0.934). These results indicate that the two cohorts were well balanced and comparable, supporting the validity of subsequent model training and evaluation (Table 1).

### 3.2. Performance of Clinical-Only Models

This study first built baseline models using only three clinical variables (tumor stage, age, and sex). Among the five classifiers, SVM achieved the highest AUC of 0.52 and accuracy of 0.68, while all other models produced AUC values between 0.45 and 0.51 and accuracies between 0.58 and 0.64, indicating that clinical features alone performed barely better than random guessing.

### 3.3. Performance of Models Based on SFS-Selected Features

Using SFS, a subset of 8 radiomic features was selected and combined with clinical variables to construct predictive models. Among the five machine learning algorithms evaluated, LR achieved the best overall performance, with an accuracy of 0.72 and an AUC of 0.64 in the testing set.

**Figure 1:**
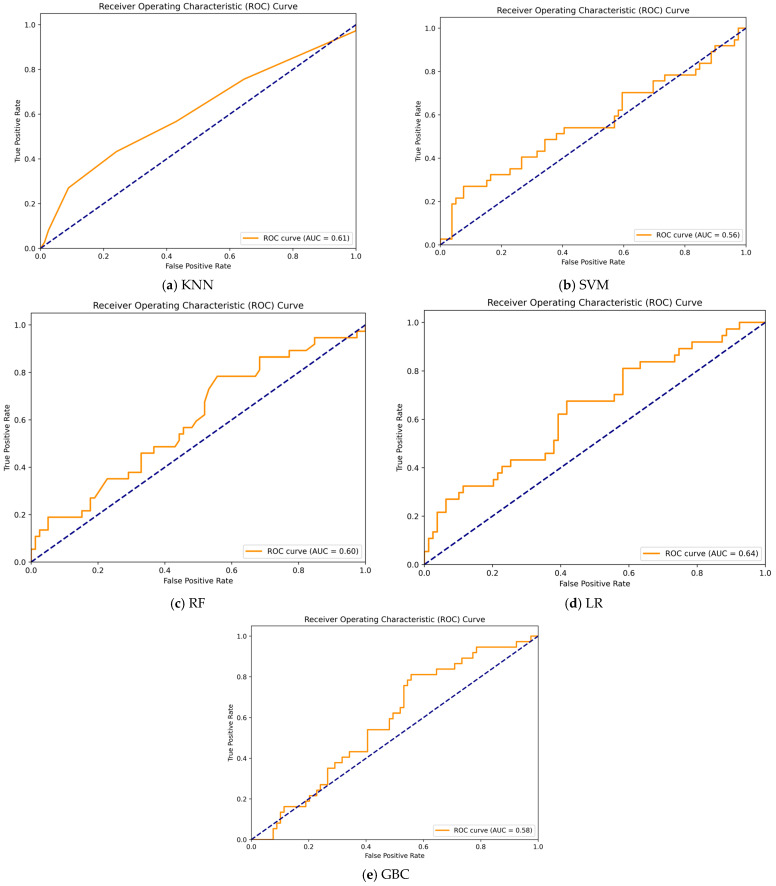
Receiver operating characteristic (ROC) curves of five machine learning models based on sequential forward selection (SFS)-selected features. (**a**) K-nearest neighbors (KNN), (**b**) support vector machine (SVM), (**c**) random forest (RF), (**d**) logistic regression (LR), and (**e**) gradient boosting classifier (GBC).

KNN showed comparable performance (accuracy = 0.71, AUC = 0.61), while SVM, RF, and GBC demonstrated relatively lower discriminative ability, with AUC values ranging from 0.56 to 0.60. Overall, LR consistently provided the most favorable balance between accuracy and discrimination under the SFS feature selection framework (Table 2, Figure 1). Specifically, the hyperparameters for LR were configured with an L2 regularization penalty (penalty = ‘l2’), a regularization strength of C = 0.02, and the ‘lbfgs’ solver, with a fixed random seed (random_state = 42) to ensure reproducibility.

### 3.4. Performance of Models Based on mRMR-Selected Features

To determine the optimal number of features for mRMR, we performed an ablation study by evaluating feature subsets of increasing size (5, 10, and 15) using all five machine learning methods. The results demonstrated that model performance plateaued at 10 radiomic features, with no significant improvement when increasing to 15 features (Table 3).

**Figure 2:**
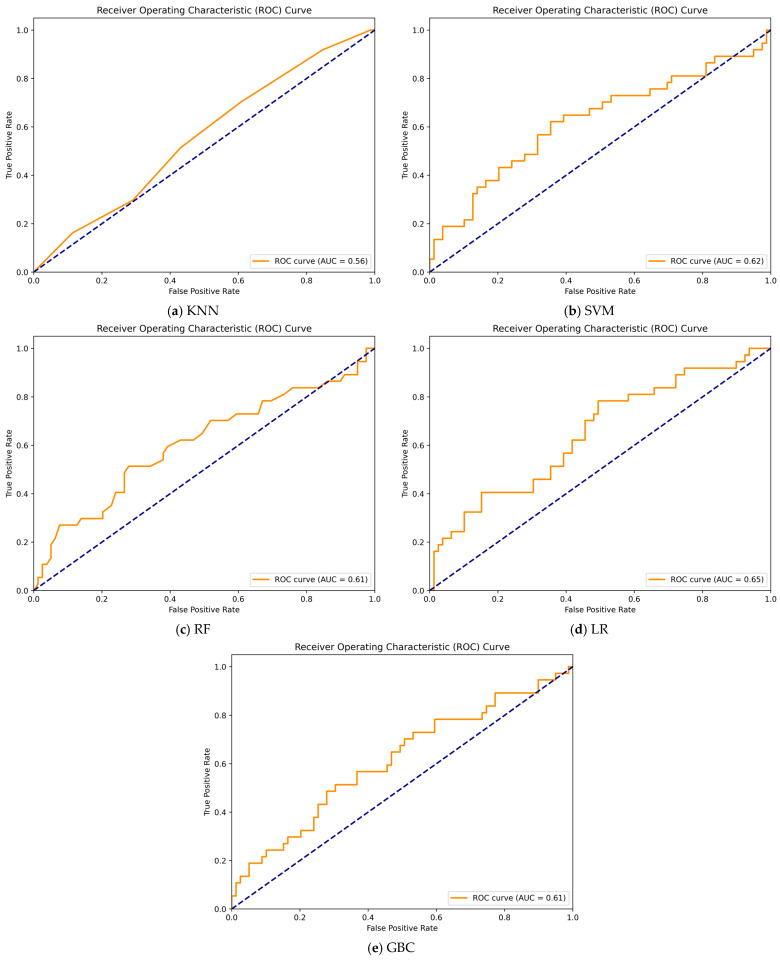
Receiver operating characteristic (ROC) curves of five machine learning models based on maximum relevance minimum redundancy (mRMR)-selected features. (**a**) K-nearest neighbors (KNN), (**b**) support vector machine (SVM), (**c**) random forest (RF), (**d**) logistic regression (LR), and (**e**) gradient boosting classifier (GBC).

Using mRMR, 10 radiomic features were selected for model construction. Among all models, LR again demonstrated the best performance, achieving an AUC of 0.65 and an accuracy of 0.72 in the testing set. Compared with SFS-based models, the mRMR-based models showed a modest improvement in AUC, suggesting that mRMR may be more effective in identifying informative and non-redundant features. SVM also showed competitive performance (AUC = 0.62), whereas KNN, RF, and GBC exhibited relatively lower AUC values. These findings suggest that mRMR combined with LR demonstrated the highest observed discrimination within this dataset, although formal statistical comparisons between models were not performed (Table 4, Figure 2). Specifically, the hyperparameters for LR were configured with an L2 regularization penalty (penalty = ‘l2’), a regularization strength of C = 0.01, and the ‘lbfgs’ solver, with a fixed random seed (random_state = 42) to ensure reproducibility.

### 3.5. Performance of Models Based on LASSO-Selected Features

**Figure 3:**
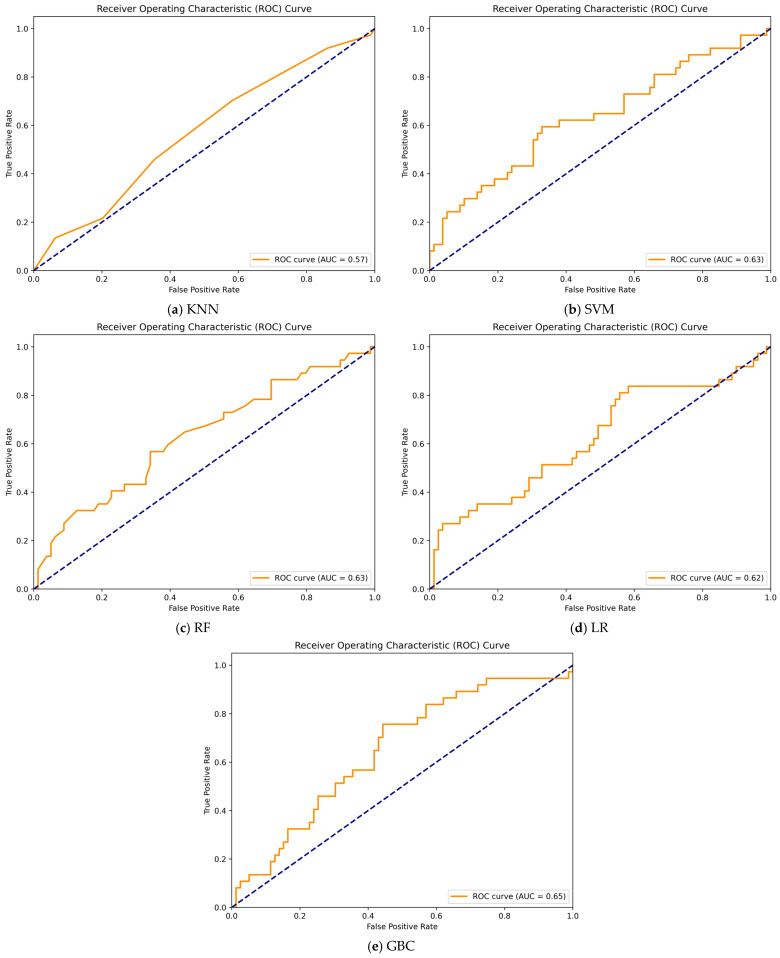
Receiver operating characteristic (ROC) curves of five machine learning models based on least absolute shrinkage and selection operator (LASSO)-selected features. (**a**) K-nearest neighbors (KNN), (**b**) support vector machine (SVM), (**c**) random forest (RF), (**d**) logistic regression (LR), and (**e**) gradient boosting classifier (GBC).

Using LASSO regression, 14 radiomic features were selected for model construction. LR achieved the highest accuracy (0.73) among all models, although its AUC (0.62) was slightly lower than that of the mRMR-LR model. Interestingly, the GBC achieved an AUC of 0.65, comparable to the best-performing mRMR-LR model, suggesting that more complex models may benefit from LASSO-based feature selection. However, LR maintained superior overall stability in terms of accuracy across different feature selection strategies. These results highlight the trade-off between model complexity and generalizability in radiomics-based prediction (Table 5, Figure 3). Specifically, the hyperparameters for LR were configured with an L2 regularization penalty (penalty = ‘l2’), a regularization strength of C = 0.02, and the ‘lbfgs’ solver, with a fixed random seed (random_state = 42) to ensure reproducibility.

### 3.6. Survival Analysis Based on SFS-Selected Features

**Figure 4:**
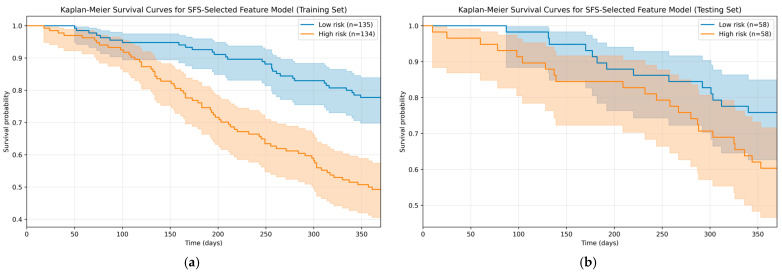
Kaplan–Meier survival curves for high- and low-risk groups based on sequential forward selection (SFS)-selected radiomic features. (**a**) Training set. (**b**) Testing set.

The proportional hazards assumption was tested. No significant violation was detected for any of the selected features (all *p* > 0.05), confirming that the Cox proportional hazards model was appropriate for this analysis. The Cox proportional hazards model was constructed using 11 SFS-selected features (Table 6). The results for Cox models are shown Table 7. The risk score for each patient was calculated based on the Cox model, and the median risk score was used as the cut-off to classify patients into high- and low-risk groups for Kaplan–Meier analysis. The model demonstrated strong risk stratification performance in the training set, with a highly significant difference between high- and low-risk groups (*p* = 5.684 × 10^−7^). In the testing set, a trend toward separation was observed (*p* = 0.074), although statistical significance was not reached. Kaplan–Meier survival curves showed clear separation between risk groups in the training cohort, while separation in the testing cohort was less pronounced. These findings suggest that SFS-based feature selection may provide good model fit in the training data but limited risk stratification ability (Figure 4).

### 3.7. Survival Analysis Based on mRMR-Selected Features

Although violations of the proportional hazards assumption were observed for InterquartileRange_firstorder_original and Kurtosis_firstorder_original, the Cox model was retained as an exploratory risk stratification analysis. Therefore, the resulting hazard ratio estimates should be interpreted cautiously, particularly for variables demonstrating assumption violations.

**Figure 5:**
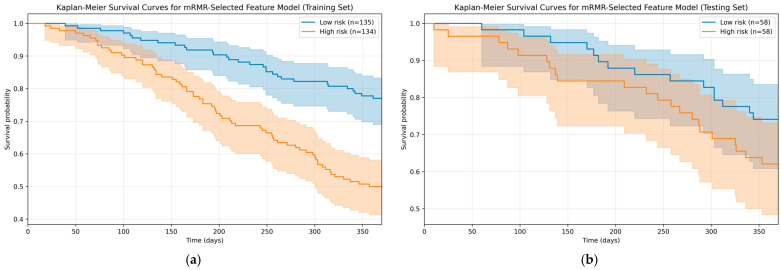
Kaplan–Meier survival curves for high- and low-risk groups based on maximum relevance minimum redundancy (mRMR)-selected radiomic features. (**a**) Training set. (**b**) Testing set.

The Cox model based on mRMR-selected features (Table 8) was constructed. The results for Cox models are shown Table 9. Patients were divided into high- and low-risk groups based on the median risk score derived from the Cox model. KM analysis demonstrated significant separation between high- and low-risk groups in the training set (*p* = 2.366 × 10^−6^). However, this separation was not statistically significant in the testing set (*p* = 0.148). Although Kaplan–Meier curves indicated a general trend toward poorer survival in the high-risk group, the reduced statistical significance suggests limited robustness in independent validation. However, several variables produced extreme hazard ratio estimates and very wide confidence intervals, indicating potential numerical instability in the Cox model. Therefore, the corresponding hazard ratio estimates and their prognostic implications should be interpreted cautiously. These results indicate that while mRMR improves feature relevance for classification tasks, its performance in survival modeling may be less consistent (Figure 5).

### 3.8. Survival Analysis Based on LASSO-Selected Features

The proportional hazards assumption was tested. No significant violation was detected for any of the selected features (all *p* ≥ 0.05), confirming that the Cox proportional hazards model was appropriate for this analysis.

**Figure 6:**
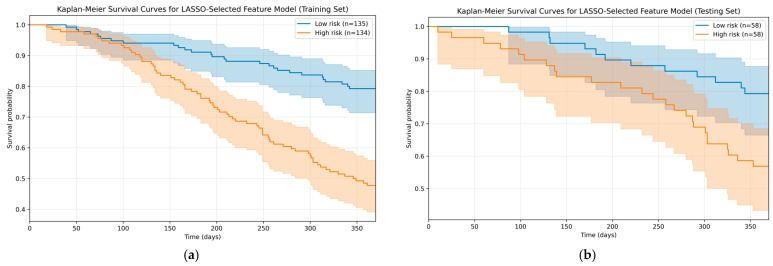
Kaplan–Meier survival curves for high- and low-risk groups based on least absolute shrinkage and selection operator (LASSO)-selected radiomic features. (**a**) Training set. (**b**) Testing set.

The Cox model constructed using LASSO-selected features (Table 10) demonstrated the best overall performance in survival analysis. The results for Cox models are shown Table 11. Patients were divided into high- and low-risk groups based on the median risk score derived from the Cox model. Significant separation between high- and low-risk groups was observed in both the training set (*p* = 8.346 × 10^−8^) and the testing set (*p* = 0.0095). Kaplan–Meier curves showed clear and consistent divergence between risk groups across both cohorts, indicating good internal validation performance of the risk stratification model. These findings suggest that LASSO-based feature selection may be particularly suitable for survival modeling, likely due to its embedded regularization mechanism that reduces overfitting (Figure 6).

## 4. Discussion

In recent years, machine learning and deep learning have emerged as powerful tools for prognostic modeling in non-small cell lung cancer. Conventional ML approaches [21] have been widely applied to radiomics features extracted from CT images for survival prediction, metastasis prediction [22] and risk stratification. More recently, deep learning methods have demonstrated remarkable capabilities in multimodal data fusion (integrating imaging, clinical, and genomic data) [23,24], automated tumor segmentation [25], and precision diagnosis [23]. However, unlike deep learning models, which are often considered “black boxes” with limited interpretability [26], machine learning approaches provide more transparent and clinically interpretable predictions, allowing clinicians to identify key predictive features and understand the rationale behind each decision.

In this study, we systematically evaluated the impact of different feature selection strategies and machine learning algorithms on CT radiomics-based prediction of 12-month overall survival in patients with non-small cell lung cancer. Several important findings emerged from our analysis, providing methodological insights into radiomics-based prognostic model development.

First, logistic regression consistently demonstrated stable performance across all feature selection strategies. Despite the increasing popularity of complex machine learning models, such as random forest and gradient boosting, our results suggest that simpler linear models may achieve more stable performance in high-dimensional radiomics datasets with limited sample sizes. This finding is consistent with previous radiomics studies, which have reported that simpler models often outperform more complex algorithms due to reduced overfitting in small-sample, high-dimensional scenarios [27]. For example, several studies have shown that logistic regression achieves comparable or even superior performance compared with ensemble methods when the number of features is large relative to the sample size [28]. These results further support the notion that model simplicity may be advantageous in high-dimensional radiomics analysis. In particular, prior work based on the NSCLC radiomics dataset has demonstrated that model performance is highly sensitive to feature selection and model complexity, and that linear classifiers can provide competitive or superior performance compared to non-linear models [29].

Second, the choice of feature selection method had a noticeable impact on model performance. Among the evaluated methods, mRMR achieved the highest observed AUC among the evaluated models. However, given the relatively small differences in performance between competing models, these findings should be interpreted descriptively rather than as evidence of statistically superior performance. Similar findings have been reported in previous studies, where mRMR has been shown to improve model interpretability and stability by reducing feature redundancy [30]. In contrast, LASSO-based feature selection showed favorable performance in survival analysis, achieving significant survival separation in both the training and testing cohorts. This observation is in line with previous prognostic studies, where LASSO has been widely used for feature selection in Cox regression models due to its ability to handle multicollinearity and reduce overfitting [31]. Therefore, LASSO may represent a useful feature selection strategy for survival modeling in radiomics datasets. This may be attributed to the embedded regularization mechanism of LASSO, which effectively reduces overfitting while preserving the most predictive features [32].

Third, a discrepancy was observed between classification performance and survival analysis results. While mRMR-based models achieved the highest discriminative ability in predicting 12-month survival status, only LASSO-based Cox models demonstrated favorable performance in risk stratification. This finding highlights that optimal feature selection strategies may differ depending on the modeling objective and emphasizes the importance of aligning feature selection methods with downstream analytical tasks. Similar task-dependent behavior has been noted in previous machine learning studies, suggesting that classification and survival modeling may require distinct optimization strategies [33].

Importantly, the overall predictive performance of the classification models was moderate (AUC around 0.60–0.65), which is consistent with previous radiomics studies reporting methodological limitations [5]. For example, the widely cited study by Aerts et al. [27] demonstrated that while radiomic features capture tumor phenotype, their predictive performance remains limited when used in isolation. These findings suggest that although CT radiomics captures information related to tumor heterogeneity, its standalone predictive ability may be insufficient to provide robust prognostic discrimination or support clinical decision-making [34]. Therefore, CT radiomics alone is unlikely to fully capture the complexity of patient outcomes. Future studies incorporating multi-modal data sources, such as clinical, genomic, pathological, or deep learning-derived features, may further improve predictive performance and should be explored in larger externally validated cohorts [35].

Several limitations of this study should be acknowledged. First, analysis was restricted to a single publicly available dataset without external validation, which limits generalizability across imaging protocols and patient populations. In addition, patients censored before the 12-month endpoint were excluded from the classification analyses. Although this approach ensured a clearly defined outcome label, it may have introduced selection bias because excluded patients may differ systematically from those retained in the final cohort. Second, only a limited number of clinical variables (tumor stage, sex, and age) were available, and the testing cohort was relatively small, which may have constrained model performance and increased uncertainty in performance estimates. Furthermore, model evaluation was based on a single random train–test split without repeated resampling procedures, and therefore the reported performance metrics may be influenced by variability in cohort allocation. Third, scanner and protocol variability are a recognized challenge in radiomics. Our dataset included CT images acquired using different scanners and imaging protocols, and image preprocessing was applied to improve feature robustness. However, these measures cannot completely eliminate batch effects. Consequently, the present findings should be interpreted primarily as a methodological comparison rather than evidence of a clinically validated prognostic model. In addition, calibration metrics were not evaluated in the present study because the primary objective was methodological comparison rather than individualized risk estimation. Future studies aimed at clinical implementation should incorporate calibration assessment, including calibration curves and Brier scores, together with external validation. Furthermore, formal pairwise statistical comparisons between competing classification models were not performed; therefore, small differences in AUC should be interpreted with caution. Violations of the proportional hazards assumption were observed in the mRMR-based Cox model, and the corresponding survival analysis findings should therefore be regarded as exploratory and require further validation in larger independent cohorts. Numerical instability was also observed in the mRMR-based Cox model, as reflected by extreme hazard ratio estimates and very wide confidence intervals for several variables. Therefore, the corresponding hazard ratio estimates and prognostic implications should be interpreted cautiously.

In conclusion, this study demonstrates that both feature selection strategy and model choice play critical roles in radiomics-based survival prediction. Simpler models such as logistic regression may provide relatively stable performance within the studied dataset, while LASSO-based approaches may be more suitable for survival modeling. These findings provide methodological guidance for future radiomics model development and validation studies in NSCLC. To our knowledge, this is the first comparison of SFS, mRMR, and LASSO feature selection methods combined with multiple machine learning algorithms for radiomics-based survival prediction in NSCLC, providing valuable methodological insights for the field.

## 5. Conclusions

In this study, we systematically evaluated the impact of feature selection strategies and machine learning algorithms on CT radiomics-based prediction of 12-month overall survival in patients with non-small cell lung cancer. The results demonstrated that predictive performance was influenced by both feature selection methods and algorithm choice. Logistic regression achieved the most stable classification performance across feature selection strategies, whereas LASSO-based approaches showed favorable performance for survival risk stratification. Overall, this study provides methodological insights into radiomics-based prognostic modeling and offers practical guidance for future model development and validation studies in NSCLC. However, because validation was limited to a single publicly available dataset without external confirmation, the findings should be interpreted as methodological evidence rather than as a clinically validated prognostic tool.

## Figures and Tables

**Table 1:** Baseline Characteristics of Training and Testing Cohorts.

	Training Set (*n* = 269)	Testing Set (*n* = 116)	*p*-Value
**Age**			0.343
**Clinical T Stage**			0.791
T1	60	29	
T2	100	37	
T3	34	15	
T4	75	35	
**Clinical N Stage**			0.177
N0	103	51	
N1	18	2	
N2	94	37	
N3	54	26	
**Clinical M Stage**			0.320
M0	265	116	
M1	4	0	
**Overall Stage**			0.876
1	57	26	
2	28	9	
3	72	31	
4	112	50	
**Sex**			0.934
Male	186	79	
Female	83	37	

**Table 2:** Performance comparison of five machine learning models using sequential forward selection (SFS)-selected radiomic features.

Model	Accuracy (CI)	AUC (CI)	Precision	Recall	F1 Score	Specificity
LR	0.72 (0.63, 0.80)	0.64 (0.52, 0.74)	0.64	0.24	0.35	0.94
KNN	0.71 (0.62, 0.79)	0.61 (0.49, 0.71)	0.59	0.27	0.37	0.91
SVM	0.69 (0.61, 0.77)	0.56 (0.44, 0.68)	1	0.03	0.05	1
RF	0.64 (0.55, 0.73)	0.60 (0.48, 0.71)	0.42	0.35	0.38	0.77
GBC	0.60 (0.51, 0.69)	0.58 (0.46, 0.68)	0.34	0.27	0.30	0.76

AUC: area under the receiver operating characteristic curve; GBC: gradient boosting classifier; KNN: k-nearest neighbors; LR: logistic regression; RF: random forest; SVM: support vector machine.

**Table 3:** Ablation study results for mRMR feature selection.

	LR	KNN	SVM	RF	GBC
**Top 5 Radiomic features**					
AUC	0.64	0.54	0.60	0.65	0.60
Accuracy	0.70	0.61	0.68	0.69	0.63
**Top 10 Radiomic features**					
AUC	0.65	0.56	0.62	0.61	0.61
Accuracy	0.72	0.66	0.71	0.65	0.63
**Top 15 Radiomic features**					
AUC	0.65	0.58	0.62	0.63	0.59
Accuracy	0.72	0.66	0.69	0.70	0.63

**Table 4:** Performance comparison of five machine learning models using maximum relevance minimum redundancy (mRMR)-selected radiomic features.

Model	Accuracy (CI)	AUC (CI)	Precision	Recall	F1 Score	Specificity
LR	0.72 (0.64, 0.81)	0.65 (0.53, 0.76)	0.78	0.19	0.30	0.97
KNN	0.66 (0.57, 0.74)	0.56 (0.44, 0.65)	0.40	0.16	0.23	0.89
SVM	0.71 (0.62, 0.79)	0.62 (0.50, 0.74)	0.80	0.11	0.19	0.99
RF	0.65 (0.56, 0.73)	0.61 (0.48, 0.72)	0.43	0.32	0.37	0.80
GBC	0.63 (0.54, 0.72)	0.61 (0.49, 0.72)	0.41	0.35	0.38	0.76

AUC: area under the receiver operating characteristic curve; GBC: gradient boosting classifier; KNN: k-nearest neighbors; LR: logistic regression; RF: random forest; SVM: support vector machine.

**Table 5:** Performance comparison of five machine learning models using least absolute shrinkage and selection operator (LASSO)-selected radiomic features.

Model	Accuracy (CI)	AUC (CI)	Precision	Recall	F1 Score	Specificity
LR	0.73 (0.65, 0.81)	0.62 (0.50, 0.74)	0.71	0.27	0.39	0.95
KNN	0.68 (0.60, 0.77)	0.57 (0.47, 0.67)	0.50	0.14	0.21	0.94
SVM	0.69 (0.61, 0.77)	0.63 (0.50, 0.74)	1.0	0.03	0.05	1.0
RF	0.66 (0.58, 0.75)	0.63 (0.51, 0.74)	0.46	0.35	0.40	0.81
GBC	0.66 (0.58, 0.75)	0.65 (0.53, 0.75)	0.46	0.32	0.38	0.82

AUC: area under the receiver operating characteristic curve; GBC: gradient boosting classifier; KNN: k-nearest neighbors; LR: logistic regression; RF: random forest; SVM: support vector machine.

**Table 6:** SFS-selected features.

Category	Feature Name
Clinical feature	Tumor stage
	Sex
	Age
Radiomic feature	SurfaceVolumeRatio_shape_original
	MeanAbsoluteDeviation_firstorder_original
	Range_firstorder_original
	DifferenceVariance_glcm_original
	Idm_glcm_original
	InverseVariance_glcm_original
	LargeDependenceEmphasis_gldm_original
	ShortRunEmphasis_glrlm_original

**Table 7:** Results for Cox model based on SFS-selected features.

Features	HR (95% CI)	*p* Value
Tumor stage	1.12 (0.92–1.37)	0.26
Sex	1.46 (0.90–2.37)	0.13
Age	1.29 (1.04–1.61)	0.02
SurfaceVolumeRatio_shape_original	1.39 (0.81–2.39)	0.23
MeanAbsoluteDeviation_firstorder_original	1.17 (0.64–2.13)	0.60
Range_firstorder_original	1.11 (0.92–1.35)	0.28
DifferenceVariance_glcm_original	0.81 (0.50–1.30)	0.38
Idm_glcm_original	0.01 (0.00–5.86)	0.16
InverseVariance_glcm_original	9.36 (0.34–255.54)	0.18
LargeDependenceEmphasis_gldm_original	25.67 (0.22–2944.36)	0.18
ShortRunEmphasis_glrlm_original	0.93 (0.19–4.43)	0.93

**Table 8:** mRMR-selected features.

Category	Feature Name
Clinical feature	Tumor stage
	Sex
	Age
Radiomic feature	DifferenceEntropy_glcm_original
	MCC_glcm_original
	MajorAxisLength_shape_original
	Median_firstorder_original
	JointEnergy_glcm_original
	VoxelVolume_shape_original
	Mean_firstorder_original
	Kurtosis_firstorder_original
	MeshVolume_shape_original
	InterquartileRange_firstorder_original

**Table 9:** Results for Cox model based on mRMR-selected features.

Features	HR (95% CI)	*p* Value
Tumor stage	1.01 (0.83–1.21)	0.95
Sex	1.03 (0.66–1.62)	0.88
Age	1.03 (0.83–1.28)	0.81
DifferenceEntropy_glcm_original	1.13 (0.53–2.41)	0.75
MCC_glcm_original	1.04 (0.81–1.32)	0.77
MajorAxisLength_shape_original	1.01 (0.76–1.34)	0.94
Median_firstorder_original	1.05 (0.48–2.27)	0.90
JointEnergy_glcm_original	1.04 (0.69–1.56)	0.84
VoxelVolume_shape_original	0.00 (0.00–inf)	0.83
Mean_firstorder_original	0.97 (0.31–3.01)	0.95
Kurtosis_firstorder_original	1.24 (0.79–1.95)	0.36
MeshVolume_shape_original	inf (0.00–inf)	0.83
InterquartileRange_firstorder_original	0.95 (0.54–1.66)	0.85

**Table 10:** LASSO-selected features.

Category	Feature Name
Clinical feature	Tumor stage
	Sex
	Age
Radiomic feature	Elongation_shape_original
	Flatness_shape_original
	MajorAxisLength_shape_original
	Kurtosis_firstorder_original
	Median_firstorder_original
	Minimum_firstorder_original
	JointEnergy_glcm_original
	MCC_glcm_original
	DependenceEntropy_gldm_original
	RunLengthNonUniformity_glrlm_original
	RunVariance_glrlm_original
	GrayLevelNonUniformity_glszm_original
	Contrast_ngtdm_original
	Strength_ngtdm_original

**Table 11:** Results for Cox model based on LASSO-selected features.

Features	HR (95% CI)	*p* Value
Tumor stage	1.05 (0.86–1.29)	0.61
Sex	1.38 (0.83–2.28)	0.21
Age	1.27 (1.02–1.59)	0.03
Elongation_shape_original	0.89 (0.61–1.30)	0.55
Flatness_shape_original	1.04 (0.70–1.53)	0.86
MajorAxisLength_shape_original	1.05 (0.64–1.72)	0.83
Kurtosis_firstorder_original	1.44 (1.10–1.90)	0.01
Median_firstorder_original	1.52 (0.93–2.50)	0.10
Minimum_firstorder_original	1.27 (1.01–1.61)	0.05
JointEnergy_glcm_original	0.92 (0.34–2.49)	0.88
MCC_glcm_original	1.32 (1.01–1.71)	0.04
DependenceEntropy_gldm_original	0.86 (0.58–1.27)	0.45
RunLengthNonUniformity_glrlm_original	0.97 (0.50–1.87)	0.92
RunVariance_glrlm_original	1.20 (0.58–2.47)	0.63
GrayLevelNonUniformity_glszm_original	1.27 (0.67–2.40)	0.46
Contrast_ngtdm_original	1.11 (0.73–1.69)	0.62
Strength_ngtdm_original	1.19 (0.96–1.48)	0.10

## Data Availability

Datasets were acquired from NSCLC-Radiomics from The Cancer Imaging Archive (TCIA).

## Abbreviations

The following abbreviations are used in this manuscript:

AUC	Area under the receiver operating characteristic curve
CT	Computed tomography
GBC	Gradient boosting classifier
GLCM	Gray-level co-occurrence matrix
GLDM	Gray-level dependence matrix
GLRLM	Gray-level run-length matrix
GLSZM	Gray-level size zone matrix
GTV	Gross tumor volume
HUs	Hounsfield units
KNN	k-nearest neighbors
LASSO	Least absolute shrinkage and selection operator
LR	Logistic regression
mRMR	Maximum relevance minimum redundancy
NGTDM	Neighborhood gray-tone difference matrix
NSCLC	Non-small cell lung cancer
OS	Overall survival
RF	Random forest
ROC	Receiver operating characteristic
RT	Radiotherapy
SFS	Sequential forward selection
SVM	Support vector machine
TCIA	The Cancer Imaging Archive
